# The prognostic impact of preoperative body mass index changes for patients with esophageal squamous cell carcinoma who underwent esophagectomy: A large-scale long-term follow-up cohort study

**DOI:** 10.3389/fnut.2022.947008

**Published:** 2022-11-08

**Authors:** Yi-Min Gu, Qi-Xin Shang, Han-Lu Zhang, Yu-Shang Yang, Wen-Ping Wang, Yong Yuan, Yang Hu, Guo-Wei Che, Long-Qi Chen

**Affiliations:** Department of Thoracic Surgery, West China Hospital of Sichuan University, Chengdu, China

**Keywords:** esophageal squamous cell carcinoma, body mass index changes, prognosis, nomogram, nutrition

## Abstract

**Background:**

This study aims to investigate the relationship between preoperative body mass index changes (ΔBMI) and prognosis in patients with esophageal squamous cell carcinoma who underwent esophagectomy.

**Methods:**

We identified 1,883 patients with esophageal squamous cell carcinoma who underwent curative resection in our department between January 2005 and December 2013. Patients were grouped into a stable body mass index (ΔBMI = 0) group and a decreased body mass index (ΔBMI < 0) group. Risk factors for ΔBMI were assessed using logistic regression analysis. The impact of ΔBMI on survival was investigated using Kaplan–Meier curves and Cox regression. A nomogram for survival prediction was constructed and validated.

**Results:**

The results showed that T stage (OR: 1.30, 95% CI: 1.16–1.45, *P* < 0.001) and N stage (OR: 1.24, 95% CI: 1.11–1.38, *P* < 0.001) were independent risk factors for ΔBMI. The ΔBMI < 0 group had worse overall survival than the stable body mass index group (HR: 1.25, 95% CI: 1.08–1.44, *P* = 0.002). When stratified by stage, ΔBMI had the greatest prognostic impact in stage I tumors (HR: 1.82, 95%: 1.05–3.15, *P* = 0.033). In addition, multiple comparisons showed that decreasing ΔBMI correlated with worse prognosis. The ΔBMI-based nomogram presented good predictive ability with a C-index of 0.705.

**Conclusion:**

This study demonstrates that ΔBMI < 0 had an adverse impact on the long-term survival of patients with esophageal squamous cell carcinoma undergoing esophagectomy. These results may support further investigation of preoperative nutrition support.

## Introduction

Esophageal cancer is the seventh most common malignant tumor and the sixth leading cause of cancer-related deaths worldwide (1, 2). The recently published 10-year outcome of the CROSS study reported that 49% of patients had overall disease progression in the neoadjuvant chemoradiotherapy plus surgery group (3). The prognosis for patients with esophageal cancer remains unsatisfactory. Unlike many other malignancies, esophageal cancer is more likely to cause malnutrition in patients because an obstructing tumor leads to different degrees of dysphagia. The nutritional state has been shown to be related to poor prognosis in multiple cancers (4–6). Thus, a better understanding of how the nutritional state influences cancer survival may open novel therapeutic strategies to improve cancer outcomes for patients with esophageal cancer.

Body mass index is a crucial diagnostic criterion of cancer-associated weight loss and is based on body weight and height (7). Although several studies have demonstrated the effect of preoperative body mass index (BMI) on the outcomes of patients who underwent esophagectomy for esophageal cancer (8, 9), BMI alone is not a reliable indicator of survival (10, 11). A better measure than weight alone, BMI accounts for how height might influence the effects of weight on a health response. Therefore, body mass index changes (ΔBMI) may better help to estimate the correlation between nutritional state and prognosis in various populations.

The aim of this study was to investigate the relationship between preoperative ΔBMI and prognosis in patients with esophageal squamous cell carcinoma who underwent curative esophagectomy. These findings may be helpful for the development of new potential therapeutic strategies.

## Materials and methods

### Study population

We conducted a retrospective review of our prospectively collected database to identify consecutive patients who underwent curative esophagectomy at West China Hospital of Sichuan University between January 2005 and December 2013. Eligible patients were previously diagnosed with esophageal cancer. Inclusion criteria included (1) esophageal squamous cell carcinoma; (2) receiving esophagectomy with or without neoadjuvant or adjuvant therapy; (3) R0 resection; and (4) at least 15 lymph nodes should be removed and assessed to achieve adequate nodal staging. Exclusion criteria included the coexistence of other malignancies. Ethics approval for this study was granted by the Ethics Committee of West China Hospital, Sichuan University (No. 2019641), and informed consent was waived.

### Body mass index changes measurement

BMI was defined as weight (kg) divided by height squared (m^2^). Diagnostic BMI was based on weight and height at the first visit to the outpatient clinic. BMI at 3 months before diagnosis was also recorded as the baseline BMI at the same visit, which was based on patient-reported weight and height. ΔBMI was calculated as diagnostic BMI minus baseline BMI.

### Surgery

Tumor staging was based on esophagoscopy, contrast-enhanced computed tomography of the neck, chest, and abdomen, endoscopic ultrasonography, bone scan, and magnetic resonance imaging of the brain. Standard surgery included minimally invasive esophagectomy or open thoracotomy. The surgical approach, whether minimally invasive or open, was not associated with completeness of resection. The extent of lymphadenectomy included two-field lymph node dissections and was conducted in most patients. Three-field lymph node dissections were performed in only a few patients who had suspicious cervical node disease. Postoperative patients with lymph node involvement were recommended to receive adjuvant therapy.

### Data collection and follow-up

We classified tumor stage according to the 7th edition of the TNM staging system of esophageal cancer (11). We recorded tumor recurrence, mortality, and survival status. Overall survival was measured as the time from operation to death. Patients alive or lost to follow-up were censored at the date of the last follow-up. The patients were followed up every 3 months for the first 2 years and every 6 months thereafter. Follow-up information was available over 5 years postoperatively or at the date of death.

### Statistical analysis

Statistical analyses were performed using SPSS Statistics (version 24, IBM, Armonk, NY) and the R programming language (version 3.6.3, Vienna, Austria). Normally distributed continuous variables are presented as the mean ± standard deviation (SD), non-normally distributed continuous variables as the median with interquartile range (IQR), and categorical variables as frequencies and percentages. Categorical variables were compared using the χ^2^-test, while continuous variables were analyzed by Student’s *t*-test. Risk factors were identified using Cox regression modeling. Survival analyzes were analyzed using the survival package, and Kaplan–Meier survival curves were plotted using the survminer package in R. We used the rsm package in R to develop the prediction model. For multiple comparisons, the *P*-value was adjusted using the Benjamini and Hochberg method by the fdrtool package in R. A bootstrap with 1,000 resamples was used to perform internal validation. The C-index was used to measure the prediction performance. Calibration was assessed by using the Hosmer–Lemeshow goodness-of-fit test (12). Statistical significance was set at a two-sided *P*-value less than 0.05.

## Results

### Basic characteristics and risk factors for ΔBMI

Of 1,883 eligible patients, 1,162 patients (61.7%) were grouped into the ΔBMI = 0 group, and 721 patients (38.3%) were grouped into the ΔBMI < 0 group. No patient had an increased body mass index in the ΔBMI < 0 group. The characteristics of the ΔBMI = 0 and ΔBMI < 0 groups are summarized in Table 1. There was no significant difference in baseline BMI between the two groups (*P* = 0.083).

**TABLE 1:** Patient characteristics (*n* = 1,883).

Characteristics	Δ BMI = 0 (*n* = 1,162)	Δ BMI < 0 (*n* = 721)	*P*
Age (years)			0.360
≤55/>55	258/904	166/555	
Gender			0.355
Male/Female	953/209	597/124	
Baseline BMI	21.9 ± 2.9	22.1 ± 3.2	0.083
Tumor location			0.639
Upper/Middle/Lower	119/688/355	65/427/229	
pT stage			< 0.001
T1/T2/T3	227/216/719	78/108/535	
pN stage			< 0.001
N0/N1/N2/N3	676/293/155/38	338/201/127/55	
Differentiation			0.010
High/Moderate/Low	193/692/277	83/454/184	
LVI			0.221
No/Yes	1,111/51	683/38	
Surgical approach			0.914
Open/Minimal/Hybrid	1,105/15/42	684/11/26	
Adjuvant therapy			0.773
No/Yes	700/462	431/290	

ΔBMI, body mass index changes; BMI, body mass index; LVI, lymphovascular invasion.

On univariate analysis, T stage (OR: 1.385, 95% CI: 1.249–1.535, *P* < 0.001), N stage (OR: 1.348, 95% CI: 1.216–1.495, *P* < 0.001), and differentiation (OR: 1.195, 95% CI: 1.027–1.390, *P* = 0.021) were identified as potential risk factors and included in multivariate analysis (Table 2). On multivariate analysis, our results indicated that T stage (OR: 1.296, 95% CI: 1.163–1.445, *P* < 0.001) and N stage (OR: 1.241, 95% CI: 1.113–1.383, *P* < 0.001) were independent risk factors for body mass index changes (Table 2).

**TABLE 2:** Risk factors for body mass index changes identified by logistic regression.

Variables	Univariate analysis			Multivariate analysis		
	OR	95% CI	*P*	OR	95% CI	*P*
T stage	1.39	1.25–1.53	< 0.001	1.30	1.16–1.45	< 0.001
N stage	1.35	1.22–1.50	< 0.001	1.24	1.11–1.38	< 0.001
Differentiation	1.20	1.03–1.40	0.021	1.06	0.91–1.25	0.443

OR, odds ratio; CI, confidence interval.

### ΔBMI and prognosis in different stages

**FIGURE 1:**
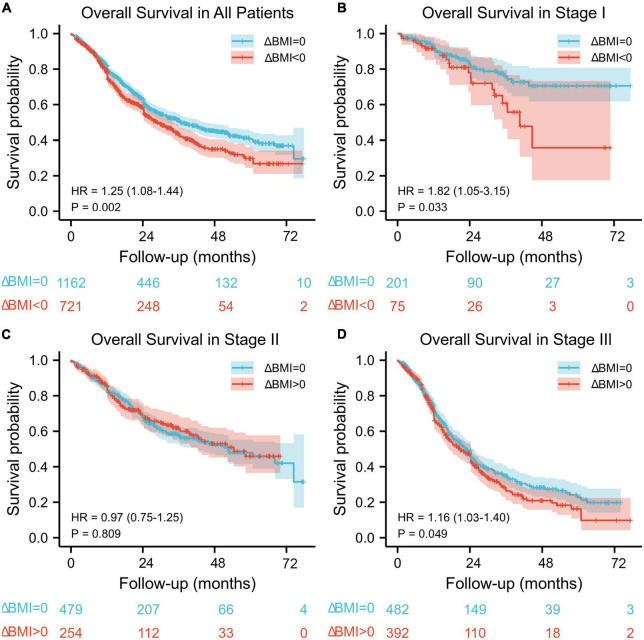
Kaplan–Meier overall survival curve according to preoperative body mass index changes for all patients **(A)** and for stage I **(B)**, stage II **(C)**, and stage III **(D)** patients.

The median follow-up for the entire cohort was 34.6 months (95% CI: 32.8–36.4) using the reverse Kaplan–Meier method. The median overall survival was 36 months (95% CI: 32.4–44.4) among 1,162 patients without body mass index changes and 28.8 months (95% CI: 25.2–33.6) among 721 patients with body mass index changes. There was a significant difference in overall survival between these two groups (HR: 1.25, 95% CI: 1.08–1.44, *P* = 0.002) (Figure 1A).

When stratified by stage, there was a significant difference in overall survival between the ΔBMI = 0 group and ΔBMI < 0 group with stage I (HR: 1.82, 95%: 1.05–3.15, *P* = 0.033) and stage III (HR: 1.16, 95%: 1.03–1.40, *P* = 0.049) disease (Figures 1B,D), but no significant difference was found in stage II (HR: 0.97, 95%: 0.75–1.25, *P* = 0.809) (Figure 1C).

### Determination of the optimal ΔBMI cutoff

**FIGURE 2:**
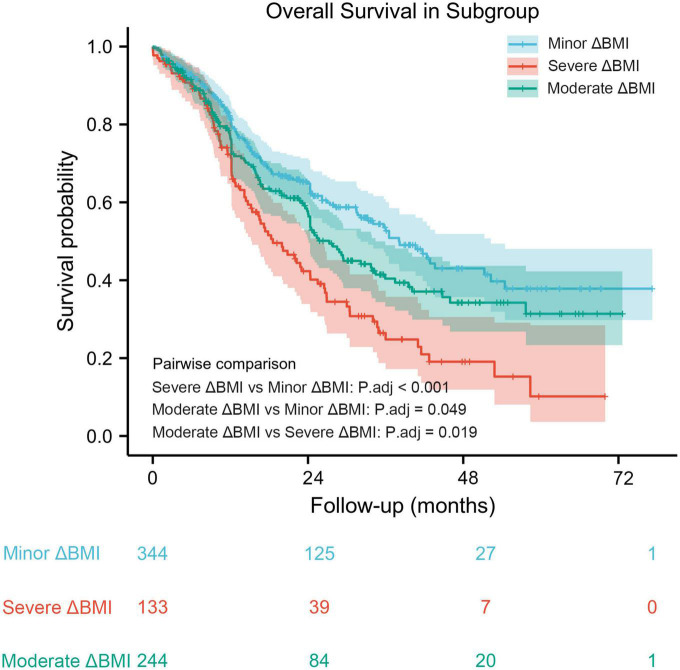
A preoperative body mass index changes-based nomogram for predicting prognosis after curative resection of esophageal squamous cell carcinoma.

**FIGURE 3:**
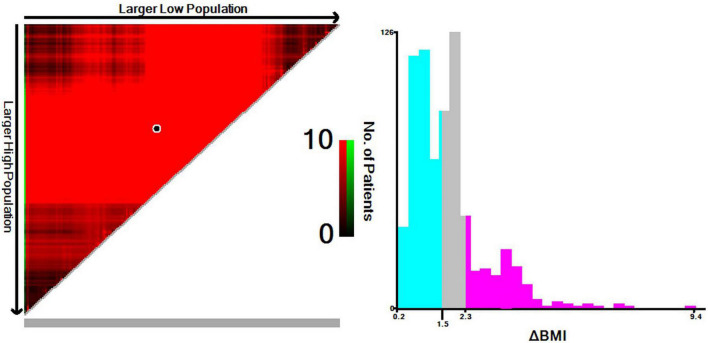
Kaplan–Meier overall survival curve stratified by optimal cutoff points in patients with decreased body mass index.

Based on the results from the X-tile program, the optimal cutoff points for overall survival were determined to be −1.5 and −2.3 kg/m^2^ (Figure 2). Accordingly, patients with 0 > ΔBMI ≥ −1.5 kg/m^2^ were defined as the minor ΔBMI group, −1.5 kg/m^2^ > ΔBMI ≥ −2.3 kg/m^2^ moderate ΔBMI group, and ΔBMI < −2.3 kg/m^2^ severe ΔBMI group. Then, we validated the cutoff points for survival stratification. The results of the Kaplan–Meier overall survival curve showed that decreasing BMI changes were associated with worse prognosis (severe ΔBMI vs. minor ΔBMI, adjusted *P* < 0.001; severe ΔBMI vs. moderate ΔBMI, adjusted *P* = 0.019; moderate ΔBMI vs. minor ΔBMI, adjusted *P* = 0.049) (Figure 3).

### Establishment of a model

Variables with statistical significance in univariate analysis were entered into multivariate analysis. Multivariate Cox regression models determined that T stage (HR: 1.325, 95% CI: 1.210–1.451; *P* < 0.001), N stage (HR: 1.206, 95% CI: 1.114–1.306; *P* < 0.001), ΔBMI (HR: 1.174, 95% CI: 1.014–1.358; *P* = 0.032), differentiation (HR: 1.134, 95% CI: 1.001–1.284; *P* = 0.047), presence of lymphovascular invasion (HR: 1.415, 1.034–1.936; *P* = 0.03), and adjuvant therapy (HR: 0.819, 95% CI: 0.768–0.874; *P* < 0.001) were significant predictors of overall survival (Table 3).

**TABLE 3:** Cox regression analyses of survival for 1,883 patients with esophageal cancer.

Variables	Univariate analysis			Multivariate analysis		
	HR	95% CI	*P*	HR	95% CI	*P*
Gender	0.78	0.64–0.96	0.016	0.91	0.73–1.12	0.353
Age	1.23	1.03–1.48	0.02	1.11	0.93–1.33	0.256
ΔBMI	1.38	1.19–1.59	< 0.001	1.17	1.01–1.36	0.032
Tumor location	0.98	0.87–1.11	0.795	–	–	−
T stage	1.53	1.41–1.67	< 0.001	1.33	1.21–1.45	< 0.001
N stage	1.51	1.40–1.62	< 0.001	1.21	1.11–1.31	< 0.001
Differentiation	1.25	1.11–1.40	< 0.001	1.13	1.00–1.28	0.047
LVI	1.38	1.01–1.89	0.043	1.42	1.03–1.94	0.030
Surgical approach	1.03	0.86–1.24	0.713	–	–	−
Adjuvant therapy	0.93	0.85–0.99	0.028	0.82	0.77–0.87	< 0.001

ΔBMI, body mass index changes; LVI, lymphovascular invasion; HR, hazard ratio; CI, confidence interval.

**FIGURE 4:**
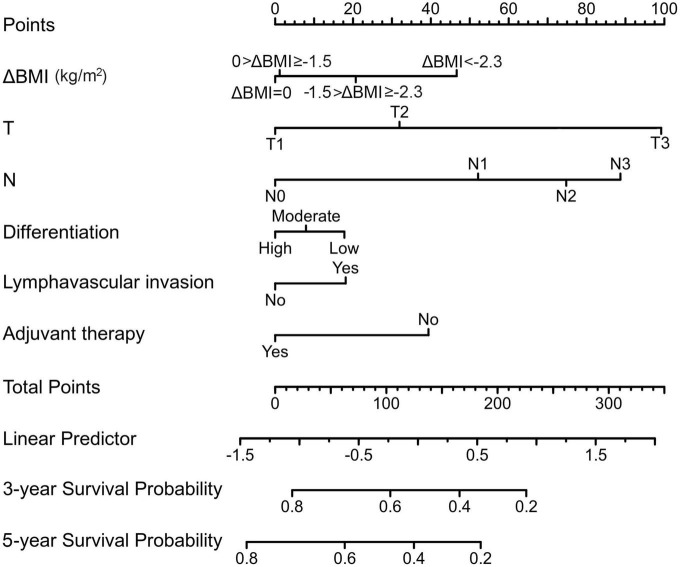
A preoperative body mass index changes-based nomogram for predicting prognosis after curative resection of esophageal squamous cell carcinoma.

**FIGURE 5:**
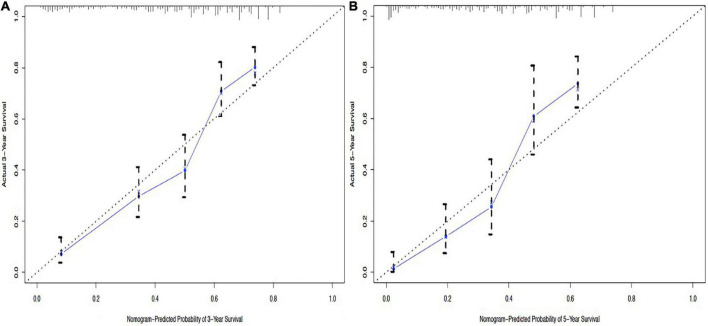
Calibration curves of the nomogram to predict 3-year overall survival **(A)** and 5-year overall survival **(B)**.

From this model, we developed a ΔBMI-based nomogram to predict the prognosis for patients with esophageal squamous cell carcinoma who underwent surgery (Figure 4). The Harrell C-index for the predictive nomogram was 0.706 (95% CI, 0.693–0.721) in the training cohort, which was 0.710 by bootstrapping validation. The nomogram calibration plot indicates that the nomogram was well calibrated, with mean predicted probabilities for each subgroup close to observed probabilities (Figure 5). The Hosmer–Lemeshow goodness-of-fit test *P*-value for the logistic regression model was not significant, indicating a good model fit.

## Discussion

In this large-scale, long-term follow-up, retrospective, cohort study, we found that patients with ΔBMI = 0 had a median overall survival of approximately 7 months longer than the ΔBMI < 0 group. Several studies have revealed that preoperative weight loss was associated with worse long-term prognosis (13–15). Rather than measuring weight changes alone, BMI also takes into account how height might affect an individual’s health. However, few studies have focused on the impact of body mass index changes on the survival of esophageal squamous cell carcinoma patients. Loehrer et al. (16) found that BMI gain compared with average adult BMI was associated with poor esophageal adenocarcinoma survival. However, the definition of BMI changes was quite different from the current study, and the sample size was too small (285 patients) to make robust conclusions.

In the subgroup analysis, we demonstrated that ΔBMI was a strong predictor of poor prognosis for stage I disease. In contrast, ΔBMI has less prognostic impact for stage III disease. Unexpectedly, no statistical significance in mortality was found between the two groups for patients with stage II disease. These findings may be due to the inherent poor prognosis of locally advanced disease diluting the strength of ΔBMI. These results also parallel the previous detection by Zhang et al. (14), in which preoperative weight loss correlated with worse survival in patients with early-stage esophageal cancer.

In the current study, the optimal ΔBMI cutoff points were also determined. Kaplan–Meier survival analysis showed that a larger body mass index decrease was associated with worse survival. These results might warrant further investigation of preoperative nutrition support for patients with severe body mass index changes, especially in early-stage (I–II) esophageal squamous cell carcinoma.

The potential weakness might be that the impact of neoadjuvant therapy on ΔBMI was not explored. Neoadjuvant chemoradiotherapy formed the standard treatment based on the CROSS trial (17) published in 2012 and the NEOCRTEC5010 trial (18) published in 2018. However, this long-term follow-up study screened patients from 2005 to 2013, and neoadjuvant therapy was not well established in that period. Most patients enrolled in this study received adjuvant therapy. However, we would like to emphasize that it would not affect the applicability of our main conclusion. To our knowledge, few studies have reported the relationship between ΔBMI and survival outcomes during neoadjuvant therapy in esophageal cancer, except for breast, rectal, and pancreatic cancer (19–21). The predictive value of ΔBMI during neoadjuvant therapy requires further investigation in future studies.

The strengths of this study include its large scale with long-term follow-up compared with previous literature (16, 22, 23). Moreover, we evaluated whether the relationship between body mass index changes and prognosis varies with tumor stage. In addition, we found that the optimal cutoff points of body mass index changes can be used to stratify patient survival. Nomograms have long been proposed as a tool for estimating an individual risk or prognosis based on clinical variables (24). Accordingly, a body mass index changes-based nomogram was established to facilitate individualized prediction of 3- and 5-year survival probability.

There were also some limitations in our study. First, it was retrospective and observational, with inherent flaws. Second, the study population was predominantly Chinese people. Globally, esophageal squamous cell carcinoma remains the most common histological type in Asia, while adenocarcinoma is the major histology in North America and Europe (25). There are differences in body mass index between the Western and Eastern populations. Thus, the generalizability of these data to the Western population requires further validation. Finally, although the prediction accuracy of the model was validated internally, external validation was not performed.

In conclusion, ΔBMI < 0 had an adverse impact on long-term survival in patients with esophageal squamous cell carcinoma who underwent esophagectomy. The earlier the tumor stage was, the greater the impact. In addition, decreasing ΔBMI was associated with worse prognosis. These results might warrant further investigation of preoperative nutrition support, especially in early cancer with severe ΔBMI.

## Data availability statement

The original contributions presented in this study are included in the article/supplementary material. Further inquiries can be directed to the corresponding author/s.

## Ethics statement

The studies involving human participants were reviewed and approved by the Ethics Committee of West China Hospital, Sichuan University. The Ethics Committee waived the requirement of written informed consent for participation.

## Author contributions

L-QC and Y-MG conceptualized the study, revised the manuscript, and supervised the study. Y-MG and Q-XS collected the data, drafted the manuscript, and made the figures. H-LZ, Y-SY, YY, YH, and G-WC revised the manuscript. All authors read and approved the final manuscript.
